# Post-Aspiration Balloon Reinjection During Urethral Catheter Removal in Adult Men and Women: A Systematic Review and Meta-Analysis of Randomized and Quasi-Randomized Trials

**DOI:** 10.3390/healthcare14182974

**Published:** 2026-09-11

**Authors:** Jingwen Hu, Qiaoling Lin, Chunping Du, Jianmei Zhang, Tao Gao, Hongying Jiang

**Affiliations:** 1 Rehabilitation Medicine Center and Institute of Rehabilitation Medicine, West China Hospital, Sichuan University, Chengdu 610041, China; 2 Key Laboratory of Rehabilitation Medicine in Sichuan Province, West China Hospital, Sichuan University, Chengdu 610041, China; 3 Department of Gastroenterology and Hepatology, The First Affiliated Hospital of Xiamen University, School of Medicine, Xiamen University, Xiamen 361003, China

**Keywords:** balloon cuffing, balloon reinjection, device removal, hematuria, meta-analysis, pain, urethral catheters

## Abstract

**Highlights:**

**What are the main findings?**

- Evidence was drawn from 46 Chinese randomized or quasi-randomized trials involving 6395 adult men and women. Post-aspiration balloon reinjection during urethral catheter removal was associated with reductions in pain (SMD −1.14), hematuria (RR 0.21), urinary retention (RR 0.22), and catheter reinsertion (RR 0.44), as well as increased successful spontaneous voiding (RR 1.29). Certainty was low or very low, and the large pain effect was highly heterogeneous.
- No eligible English-language randomized trial was identified. This absence indicates a global evidence gap for this simple, low-cost nursing technique and supports international validation before guideline adoption.

**What are the implications of the main findings?**

- This low-cost, nurse-delivered technique remains investigational. The available evidence may justify carefully monitored evaluation in selected low-risk settings, but it is insufficient to support routine implementation or a claim of established safety.
- The geographic confinement of the evidence (all trials from China) indicates an important knowledge translation gap. International multicentre trials are needed before conclusions about effectiveness, feasibility, or safety can be transferred beyond the Chinese settings represented in the available evidence.

**Abstract:**

**Background:** Guidance on catheter removal addresses passive balloon deflation and cuffing, but the comparative effect of small-volume reinjection after complete aspiration remains uncertain. This review evaluated reinjection versus conventional complete aspiration in adult men and women. **Methods:** The review was registered in PROSPERO (CRD420261451421) after the initial search and preliminary analyses had begun. Eight English- and Chinese-language databases were formally searched from inception to 9 July 2026. Randomized and quasi-randomized controlled trials of routine transurethral catheter removal were eligible. The primary outcome was pain experienced during withdrawal of the deflated balloon through the urethra, measured during removal or at the earliest immediate post-removal assessment. Pain was pooled as Hedges’ g; a harmonized 0–10 mean difference was examined for interpretability. Secondary outcomes were pooled as risk ratios. Risk of bias was assessed with Risk of Bias 2 and certainty with GRADE. **Results:** Forty-six Chinese trials (6395 participants; both sexes represented) were included: 35 were reported as randomized and 11 used predictable quasi-random allocation. Sex-stratified outcome data were not available. Reinjection was associated with less pain (26 trials; SMD −1.14, 95% CI −1.35 to −0.92; I^2^ = 89.8%; 95% prediction interval −2.25 to −0.03); the harmonized 0–10 mean difference was −1.73 points (95% CI −2.11 to −1.35). Associations also favored reinjection for urinary retention (RR 0.22; low certainty), successful spontaneous voiding (RR 1.29; very low certainty), any hematuria (RR 0.21; very low certainty), macroscopic hematuria (RR 0.28; low certainty), microscopic hematuria (RR 0.34; very low certainty), and catheter reinsertion (RR 0.44; low certainty). Infection-related adverse events were not reported in extractable comparative form. **Conclusions:** Reinjection was associated with lower pain and fewer removal-related events compared with active complete aspiration, but certainty is low or very low, heterogeneity is substantial, all evidence is from one country, and sex-specific effects and the influence of prostatic hyperplasia or catheter diameter could not be assessed. The findings do not establish superiority over passive balloon deflation or support routine international implementation.

## 1. Introduction

Indwelling urethral catheterization is widely used in hospitals, perioperative care, rehabilitation, and long-term care. Catheter removal may be followed by pain, hematuria, urinary retention, failure to void spontaneously, and catheter reinsertion. Infection control and catheter management guidance emphasizes appropriate indications, aseptic insertion, maintenance, infection prevention, and timely removal [1,2,3,4,5,6,7,8,9,10,11,12,13,14,15,16], whereas systematic reviews and trials have focused primarily on removal timing, catheter clamping, and prophylaxis [17,18,19,20,21,22,23,24,25,26,27]. The 2024 European Association of Urology Nurses (EAUN) guideline also recommends passive balloon deflation rather than active suction and discusses the management of balloon cuffing [3]. It does not, however, recommend routine reinjection after complete aspiration. The intervention examined here should therefore be understood as a comparison with the complete aspiration technique used in the included trials, not with every alternative deflation method.

Catheter removal can itself cause mechanical trauma. After aspiration, the balloon may not collapse into a smooth cylinder; instead, folds and ridges can form a cuff around the distal catheter. In vitro studies and clinical reports indicate that cuffing depends on catheter material and deflation mechanics and can increase the effective withdrawal diameter [28,29,30,31,32,33,34]. This evidence supports a mechanical hypothesis, but most studies were laboratory investigations or reports of difficult removal and do not establish the clinical efficacy of routine reinjection.

For more than two decades, Chinese nursing teams have reported a simple modification: after complete aspiration of the balloon, 0.2–1.5 mL of sterile water, normal saline, residual aspirated fluid, or air is reinjected immediately before catheter withdrawal [35,36,37,38,39,40,41,42,43,44,45,46,47,48,49,50,51,52,53,54,55,56,57,58,59,60,61,62,63,64,65,66,67,68,69,70,71,72,73,74,75,76,77,78,79,80]. The proposed aim is to smooth the cuff and reduce friction. The evidence remains dispersed across small Chinese studies; we identified no eligible English-language randomized trial and found no previous bilingual synthesis.

Geographically variable procedural practice is not unique to catheter care. In cardiac surgery, registry data show marked between-country variation in the use of off-pump coronary artery bypass grafting and multiple arterial grafts. A global survey also found substantial regional variation in cardioplegia methods and solutions despite the absence of clear consensus [81,82]. These patterns illustrate how differences in training, local workflows, professional networks, and an incomplete comparative evidence base can sustain different procedural traditions. A country-specific evidence base therefore warrants careful assessment of transferability without either automatic dismissal or uncritical adoption.

This review had three objectives: (1) to quantify effects on pain and removal-related complications; (2) to explore whether reinjection medium, volume, study design, sample size, and clinical population explained heterogeneity; and (3) to determine whether the certainty of the current evidence is sufficient to support clinical or guideline recommendations. The review question was structured according to population, intervention, comparator, outcomes, and study design.

This meta-analysis evaluates a plausible but uncertain catheter-removal technique. It aims to define current effect estimates and their limitations, rather than treating geographically restricted, low-certainty evidence as sufficient to support routine implementation.

## 2. Materials and Methods

### 2.1. Design and Reporting

This systematic review and meta-analysis followed PRISMA 2020 [83,84]. Review work and database searching were completed on 9 July 2026, and preliminary screening and exploratory analyses were undertaken before PROSPERO registration on 15 July 2026 (CRD420261451421). The registration was therefore retrospective with respect to the initial search and preliminary analysis. During manuscript preparation, the team continued supplementary literature surveillance for potentially higher-quality studies. Complete rerun logs were not archived, so the reproducible formal search date remains 9 July 2026. Eligibility criteria, outcomes, and the primary random-effects approach were not changed after registration. The threshold for funnel-plot and Egger analyses was subsequently changed from at least 3 studies in the registry to at least 10 studies. This amendment follows conventional guidance because tests with fewer studies are underpowered and potentially misleading, and it is disclosed in Section 2.9 and the PRISMA checklist.

Generative artificial intelligence disclosure: During preparation of this revision, OpenAI Codex was used to assist in language editing. It was not used for study selection, data extraction, risk-of-bias assessment, statistical analysis, figure generation, or interpretation of new data. All AI-assisted text was reviewed and approved by the authors, who take full responsibility for the manuscript.

### 2.2. Search Strategy

Searches covered PubMed/MEDLINE, Embase (Elsevier), the Cochrane Library, Google Scholar, China National Knowledge Infrastructure, Wanfang Data, the VIP Chinese Science and Technology Journals Database, and SinoMed from inception through 9 July 2026. Supplementary File S1 reports an executable, database-specific reconstruction of the English and Chinese search strategies, including platform, fields, date coverage, and limits. Because the original line-by-line search histories and per-database exports were not supplied with the archived review materials, the reconstructed strings are not presented as verbatim contemporaneous logs. Google Scholar was supplementary; the archived log did not preserve a stable ranked-result cutoff or export depth, and this component was not described as comprehensive. The merged library retained aggregate rather than database-specific retrieval counts. Deduplication used exact identifiers when available and normalized title, author, and year matching followed by manual review of near-duplicates. These limitations are reported transparently rather than retrospectively inventing database counts.

### 2.3. Inclusion and Exclusion Criteria

#### 2.3.1. Population

Adults aged 18 years or older, including men and women, undergoing routine removal of an ordinary indwelling transurethral balloon catheter were eligible. Children, pregnant patients, and patients with suprapubic catheters, urethral reconstruction or stricture, traumatic catheterization, encrustation, or other high-risk difficult removal conditions were excluded. Mixed-population studies were eligible only when data for the routine removal population could be separated; otherwise they were excluded.

#### 2.3.2. Intervention

Complete aspiration of the original balloon contents followed by reinjection of 0.2–1.5 mL sterile water, normal saline, residual aspirated fluid, or air immediately before withdrawal, with no simultaneous pharmacological or mechanical co-intervention.

Comparator: Conventional withdrawal immediately after complete balloon aspiration, without reinjection or reinflation.

#### 2.3.3. Eligible Outcomes

The primary outcome was pain during removal. Secondary outcomes were any hematuria, macroscopic hematuria, microscopic hematuria, urinary retention, successful spontaneous voiding, and catheter reinsertion.

#### 2.3.4. Study Design

Randomized or quasi-randomized controlled trials with a concurrent conventional-removal group were eligible. Non-comparative designs, unavailable full texts, duplicate reports without additional data, studies with insufficient extractable data, partial aspiration without reinjection, unclear reinjection technique, suprapubic catheter removal, and combined interventions such as lidocaine gel, analgesics, antibiotics, or bladder clamping were excluded. Quasi-randomized trials were retained because they comprised a material part of the sparse evidence base; design was incorporated into Risk of Bias 2 judgments, GRADE downgrading, subgroup interpretation, and sensitivity analysis rather than treated as equivalent to adequately randomized trials.

### 2.4. Intervention Definition and Planned Effect Modifiers

Throughout this review, post-aspiration balloon reinjection (hereafter, reinjection) denotes complete withdrawal of the original balloon contents until no further fluid could be aspirated, followed immediately by a small, measured reinjection before gentle catheter withdrawal. The terms reinflation and re-instillation are used only when reproducing source wording or search synonyms. Medium, volume, sample size, allocation method, clinical specialty, sex, catheter material and diameter, and dwell time were prespecified as potential effect modifiers. Quantitative adjustment or meta-regression was planned only if study-level reporting was sufficiently complete and at least 10 studies contributed usable data per modeled covariate.

Experimental procedure: After complete aspiration, the specified liquid or air volume was reinjected aseptically and the catheter was withdrawn without force. Reported volumes ranged from 0.2 to 1.5 mL; most liquid protocols used 0.3–0.5 mL and most air protocols used 0.4–0.6 mL.

Control procedure: The balloon was aspirated until no further fluid could be withdrawn, after which the catheter was removed without reinjection. No additional procedural difference was eligible.

Timing and safety: Reinjection was performed immediately before withdrawal using a sterile syringe. Resistance, severe pain, suspected encrustation, urethral reconstruction or stricture, traumatic catheterization, and a history of difficult removal were not regarded as indications for reinjection and require appropriate clinical assessment.

### 2.5. Outcomes

The primary outcome was pain as the deflated balloon traversed the urethra, measured during removal or at the earliest immediate post-removal assessment. When a study reported multiple instruments at the same time point, we prioritized a validated visual analogue scale, followed by a numeric rating scale, facial scale, verbal or ordinal scale, and finally a locally adapted or unclear scale. The most complete eligible dataset was used once per comparison. Because the instruments differed, the primary synthesis used standardized mean differences calculated as Hedges’ g. Hedges’ g was preferred to Cohen’s d because many included trials were small and the correction reduces upward small-sample bias. Ordered category data were converted only when category frequencies and prespecified numeric scores were available. The mean was calculated as the frequency-weighted category score, and the standard deviation was calculated from the weighted squared deviations using an N − 1 denominator. Reconstructed data were flagged for sensitivity analysis. A separate harmonization to a 0–10 scale linearly rescaled compatible continuous instruments and was used only to aid clinical interpretation. Secondary outcomes were urinary retention, successful spontaneous voiding, any hematuria, macroscopic hematuria, microscopic hematuria, and catheter reinsertion.

### 2.6. Study Selection and Data Extraction

Two reviewers independently screened titles and abstracts, assessed full texts, and extracted all eligible outcomes and analysis inputs using a piloted form. Disagreements were resolved by discussion and, when necessary, adjudication by a third reviewer. Paired screening decisions were not retained in a format that permitted a defensible retrospective calculation of Cohen’s kappa; therefore, no agreement coefficient was reconstructed. Extracted items included the allocation method, setting, eligibility criteria, baseline age and sex when reported, catheter type, material, diameter and dwell time, intervention and comparator details, outcome definitions and timing, group sizes, and numerical results. Missing items were recorded as not reported rather than inferred.

### 2.7. Risk-of-Bias Assessment

Two reviewers independently applied the Risk of Bias 2 tool to five domains: the randomization process, deviations from intended interventions, missing outcome data, outcome measurement, and selection of the reported result. Predictable allocation based on admission order, ward, treatment route, or a comparable method was judged to confer a high risk of bias in the randomization process. Operator blinding was infeasible. For outcome measurement, low risk required an explicitly independent assessor who was unaware of allocation and used a measure unlikely to be influenced by that knowledge. Unreported assessor status was judged as some concerns; an aware assessor using an outcome plausibly influenced by allocation knowledge was judged as high risk. Participant-reported pain remained susceptible to lack of blinding even when the assessor was independent.

### 2.8. Certainty of Evidence

Two reviewers independently assessed certainty for each outcome using the Grading of Recommendations Assessment, Development and Evaluation domains of risk of bias, inconsistency, indirectness, imprecision, and publication bias. Downgrading was prespecified for serious methodological limitations, substantial unexplained heterogeneity, evidence restricted to one country or incompletely applicable populations, confidence intervals compatible with materially different effects, and funnel plot or regression evidence of small-study effects. Disagreements were resolved by consensus or third-reviewer arbitration.

### 2.9. Statistical Analysis

Continuous outcomes were pooled by inverse-variance methods as standardized mean differences (Hedges’ g) when instruments differed and as mean differences when compatible scales could be transformed to a 0–10 scale. Binary outcomes were pooled as risk ratios using the Mantel–Haenszel method. Analyses were conducted in Review Manager (RevMan version 5.3; Cochrane). Random-effects models were primary because clinical setting, catheter material and diameter, dwell time, reinjection medium and volume, allocation method, and pain measurement varied across studies. Randomized and quasi-randomized studies were combined to avoid selective exclusion from a sparse literature, but predictable allocation was treated as high risk and examined separately. When one arm had zero events, a continuity correction of 0.5 was added to each cell of the 2 × 2 table; studies with zero events in both arms did not contribute to the pooled risk ratio for that outcome. Fixed-effect reanalysis, leave-one-out analysis, and exclusion of high-risk studies were used to assess robustness. Meta-regression was not performed because catheter diameter, dwell time, sex, material, and other covariates were reported too incompletely to support stable multivariable modeling. Funnel plots and Egger regression were restricted to outcomes with at least 10 studies. This threshold superseded the three-study threshold in the initial registry entry because asymmetry tests based on fewer studies are underpowered and potentially misleading. For the three-arm Chen S 2025 trial [37], the 0.3 mL and 0.5 mL reinjection groups were combined using outcome-appropriate multi-arm formulas. The shared control group was counted once; intervention sample sizes were summed, and continuous outcome means and standard deviations were combined before pooling. This approach avoided duplicate counting of control participants.

## 3. Results

### 3.1. Study Selection

**Figure 1:**
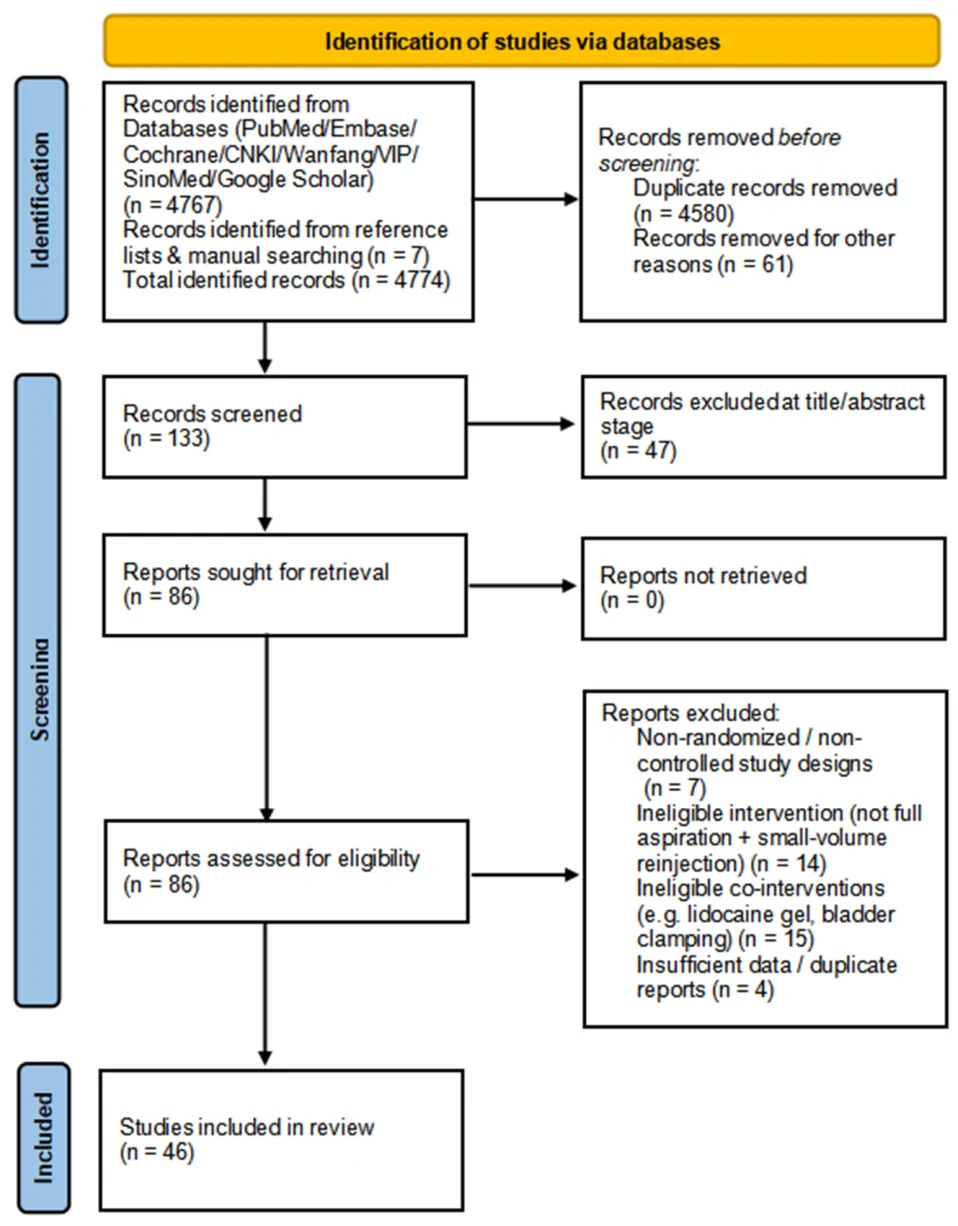
PRISMA 2020 flow diagram of study selection.

Database and manual searches identified 4774 records (4767 from databases and 7 from reference lists or manual searches). The merged export did not retain defensible per-database counts. After removal of 4580 exact or near-duplicate records and 61 records for other documented prescreening reasons, 133 records were screened; 47 were excluded. Eighty-six full texts were assessed, of which 40 were excluded: 7 were not randomized or quasi-randomized, 14 evaluated an ineligible reinjection intervention, 15 included an analgesic co-intervention during removal, and 4 had insufficient data or duplicated another report. Forty-six Chinese trials involving 6395 participants were included in the synthesis. Thirty-five reports described random allocation, although sequence generation and allocation concealment were often inadequately reported, and 11 used predictable quasi-random allocation (Figure 1).

### 3.2. Study Characteristics

The 46 studies were published between 2003 and 2025. Sample sizes ranged from 60 to 418 (median 115; interquartile range 89–186). Both men and women were represented in male-only, female-specific, and mixed clinical populations, but sex-stratified outcomes were not reported and the overall sex distribution could not be calculated reliably. Studies evaluated ordinary transurethral balloon catheters in Chinese hospitals, including two-way and, in some urological settings, three-way devices; catheter brand, material, Charriere/French diameter, balloon design, and dwell time were reported inconsistently. Thirty-six studies used a liquid, nine used air, and one did not clearly specify the medium. Most studies used 0.3–0.5 mL; others used 0.6–1.5 mL or adjusted the volume according to catheter size or dwell time. Pain instruments included visual analogue, numeric, facial, verbal or ordinal, and locally adapted or unclear scales. Because baseline and catheter covariates were missing from many studies, sex-specific pooling and adjusted meta-regression by catheter diameter or dwell time were not possible. Supplementary File S2 identifies available study-level characteristics and missing data, and Supplementary File S6 provides numerical inputs for all seven pooled outcomes. The key characteristics of the 12 selected studies are summarized in Table 1.

### 3.3. Risk of Bias

**Figure 2:**
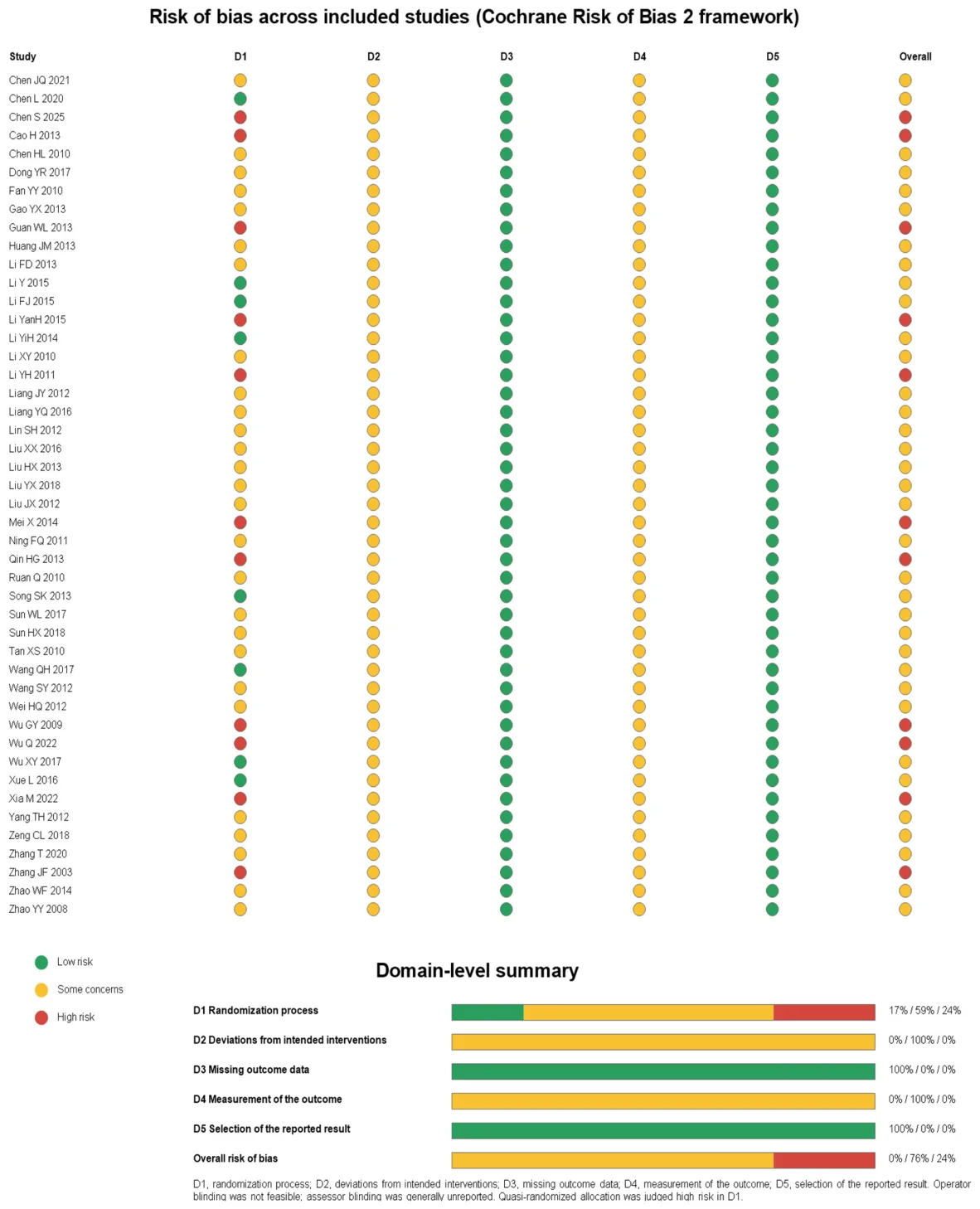
Summary plot of risk-of-bias assessments using the Cochrane Risk of Bias 2 tool for all 46 included randomized and quasi-randomized trials [35,36,37,38,39,40,41,42,43,44,45,46,47,48,49,50,51,52,53,54,55,56,57,58,59,60,61,62,63,64,65,66,67,68,69,70,71,72,73,74,75,76,77,78,79,80].

Using Risk of Bias 2, eight studies (17%) were judged at low risk for the randomization process, 27 (59%) raised some concerns, and 11 (24%) were at high risk because allocation was predictable or otherwise inadequate. All studies raised at least some concern about deviations from the intended intervention or outcome measurement because operator blinding was infeasible and blinded outcome assessment was usually not reported. Overall, 35 studies (76%) raised some concerns and 11 (24%) were at high risk; none was at low risk across all domains. Randomization and outcome measurement were therefore the principal domains that reduced confidence in the pain estimate. Excluding studies at high overall risk of bias preserved the direction of association but did not resolve heterogeneity (Section 3.4). Figure 2 presents the domain-level judgment for each study; the criteria are described in Section 2.7.

### 3.4. Primary Outcome: Pain During Catheter Removal

**Figure 3:**
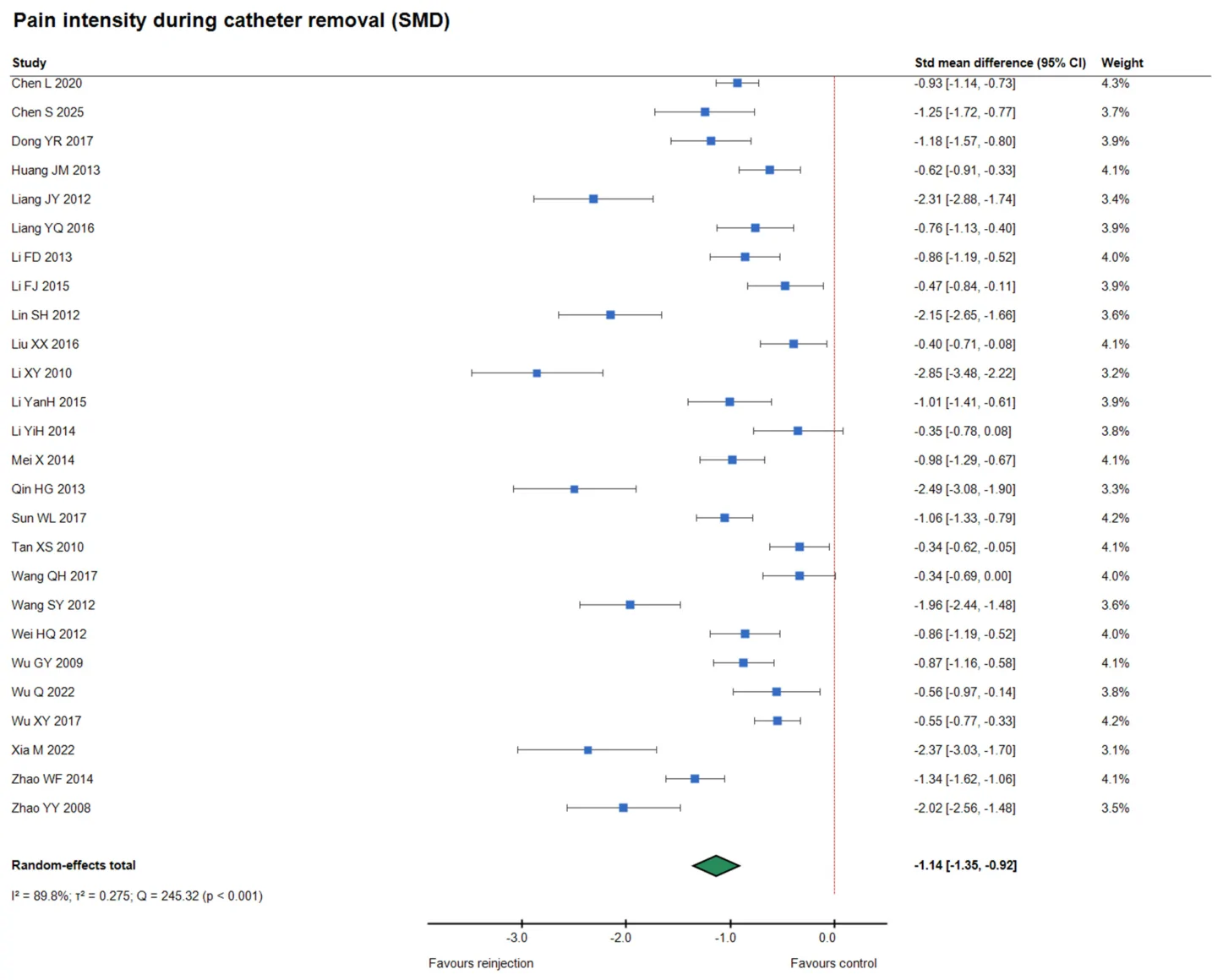
Forest plot of the effect of reinjection on pain intensity. Random-effects pooled SMD −1.14 (95% CI −1.35 to −0.92); I^2^ = 89.8%; τ^2^ = 0.275; Cochran Q = 245.32 (*p* < 0.001); 95% prediction interval −2.25 to −0.03. Blue squares denote individual-study SMDs with 95% CIs, and the green diamond denotes the pooled estimate. SMD, standardized mean difference; CI, confidence interval [36,37,40,44,45,47,48,49,50,52,53,54,55,59,61,64,66,67,68,69,70,71,74,79,80].

Twenty-six trials involving 3900 participants reported pain. Reinjection was associated with less pain than conventional removal (SMD −1.14, 95% CI −1.35 to −0.92; I^2^ = 89.8%; tau-squared = 0.275; Figure 3). The 95% prediction interval was −2.25 to −0.03, indicating that the effect in a similar future study could range from very large to close to no difference. After compatible continuous measures were harmonized to a 0–10 scale, the mean difference was −1.73 points (95% CI −2.11 to −1.35; I^2^ = 96.2%). This magnitude may be clinically noticeable, although its interpretation depends on the pain instrument and clinical context. Leave-one-out analyses preserved the direction of effect. Funnel plot asymmetry and Egger regression (*p* < 0.001) were consistent with small-study effects, publication bias, or heterogeneity.

Sensitivity analyses supported the direction of association but not the precision of the primary estimate. Excluding seven studies judged to be at high overall risk of bias yielded an SMD of −1.08 (95% CI −1.33 to −0.83; I^2^ = 90.7%; 19 studies). Leave-one-out pooled estimates ranged from −1.17 to −1.07, and fixed-effect reanalysis yielded an SMD of −0.93 (95% CI −1.00 to −0.86). Substantial heterogeneity persisted.

### 3.5. Secondary Outcomes

Associations favored reinjection for all secondary outcomes (Table 2), but urinary retention, spontaneous voiding, and catheter reinsertion are influenced by anesthesia, surgery, baseline bladder function, catheter duration, and assessment timing. They should not be interpreted as specific measures of urethral trauma. Urinary retention was less frequent (RR 0.22, 95% CI 0.14–0.32; I^2^ = 0.0%). Successful spontaneous voiding was more frequent (RR 1.29, 95% CI 1.21–1.39; I^2^ = 65.8%). Any hematuria was less frequent (RR 0.21, 95% CI 0.14–0.32; I^2^ = 52.5%). Macroscopic hematuria was less frequent (RR 0.28, 95% CI 0.19–0.41; I^2^ = 21.8%). Microscopic hematuria was less frequent (RR 0.34, 95% CI 0.22–0.52; I^2^ = 78.8%). Catheter reinsertion was less frequent (RR 0.44, 95% CI 0.28–0.69; I^2^ = 0.0%). Independent recalculation using the study-level inputs in Supplementary File S6 reproduced the study counts, participant totals, pooled effects, 95% confidence intervals, and heterogeneity statistics reported for all seven outcomes. Definitions and assessment times varied among studies. None of the included trials provided extractable comparative data on post-removal urinary tract infection or other serious adverse events. The absence of reported data must not be interpreted as evidence of safety.

### 3.6. Subgroup, Sensitivity, and Small-Study-Effect Analyses

Subgroup hypotheses for reinjection medium and volume, allocation method, sample size, specialty, sex, catheter diameter, material, and dwell time were prespecified. In the pain analysis, excluding the seven studies at high overall risk of bias retained a large association (19 studies; SMD −1.08, 95% CI −1.33 to −0.83; I^2^ = 90.7%). Both the high-risk subgroup and the remaining studies favored reinjection (SMD −1.31, 95% CI −1.74 to −0.87, and SMD −1.08, 95% CI −1.33 to −0.83, respectively), with overlapping confidence intervals. Leave-one-out pooled SMDs ranged from −1.17 to −1.07, and fixed-effect reanalysis yielded an SMD of −0.93 (95% CI −1.00 to −0.86). These analyses preserved the direction of association but did not resolve heterogeneity. The overall pain synthesis can be reconstructed from the study-level means, standard deviations, and sample sizes in Supplementary File S6. However, a quantitative sensitivity analysis excluding reconstructed ordinal category data could not be reproduced because the supplied dataset does not identify which pain records were reconstructed and the corresponding flags were not retained. We therefore do not present an unverifiable estimate. Other proposed modifiers were reported too incompletely to estimate their independent effects or perform meta-regression. Subgroup observations are therefore descriptive and should not guide treatment selection. Funnel plots and Egger regression were used only for outcomes with at least 10 studies; microscopic hematuria (nine studies) was ineligible. The pain funnel plot was asymmetric, and Egger regression was significant (*p* < 0.001).

## 4. Discussion

### 4.1. Principal Findings

This review found a consistent direction of association across pain, hematuria, urinary retention, spontaneous voiding, and catheter reinsertion. These outcomes, however, do not share a single causal pathway: urinary retention and voiding are strongly influenced by perioperative context and baseline risk, whereas pain is subjective and was measured heterogeneously. The findings are therefore compatible with reduced mechanical trauma but do not prove it. Low or very low certainty precludes firm causal, safety, or guideline-level conclusions.

### 4.2. Relationship to Existing Evidence

The most recent Cochrane review of short-term catheter removal evaluated timing and related management strategies [17]. EAUN guidance recommends passive deflation without active syringe suction to reduce cuffing and pain [3], supported by experimental evidence on deflation technique and catheter material [28,33,34]. In contrast, the included Chinese trials compared small-volume reinjection after complete aspiration with conventional complete aspiration. Their findings therefore cannot be extrapolated to passive deflation protocols, which use a different clinical comparator. This meta-analysis does not establish superiority over passive deflation, manufacturer-recommended removal, or other techniques; its contribution is to summarize a geographically localized comparison while clearly defining the comparator boundary.

This review reports where comparative trials were published. It does not establish that reinjection is practiced exclusively in China. No international survey of catheter removal technique was identified. The concentration of trials in China may reflect dissemination through local nursing journals and training networks, the ease with which a low-cost bedside modification can spread without commercial sponsorship, and a locally prevalent active aspiration comparator that differs from passive deflation guidance elsewhere. These explanations remain hypotheses because the included studies did not investigate determinants of adoption. They should not be interpreted as evidence of population-specific biology.

Comparable variation occurs in cardiac surgery. The EACTS adult cardiac surgery database reported that off-pump coronary artery bypass grafting ranged from 0.8% to 91.4%. Use of more than one arterial graft ranged from 1.2% to 76.6% across participating countries [83]. A global survey likewise found significant regional differences in cardioplegia methods, solutions, dilution, and additives, with no clear consensus and uncertain clinical consequences [84]. These examples indicate that procedures dependent on operator training and local routines can diverge widely when definitive comparative evidence is unavailable. The Chinese evidence should not be dismissed, but it should also not be generalized internationally. The effect should instead be tested across catheter products, comparator techniques, clinical settings, and health systems.

### 4.3. Mechanistic Insights from Effect-Size Patterns

The pattern of findings is compatible with an injury–pain hypothesis, but the mechanistic evidence is indirect. Laboratory studies show that active aspiration, catheter material, and incomplete elastic recovery can produce a cuff [28,33,34]. A small-volume reinjection might redistribute balloon material and blunt a ridge. However, the included trials did not directly measure balloon geometry, withdrawal force, mucosal injury, or neural activation. Pain and hematuria therefore cannot be assumed to arise solely from cuffing, and urinary retention or catheter reinsertion cannot be attributed solely to reduced urethral trauma.

### 4.4. Clinical Implications

Given the low or very low certainty of evidence, current evidence does not support routine clinical adoption. At most, the technique could be evaluated under a prospectively defined research or local quality improvement protocol for selected adults undergoing uncomplicated short-term transurethral catheter removal. Such protocols should record catheter type, deflation method, reinjection medium and volume, resistance, pain, bleeding, retention, and reinsertion. Reinjection must not be used to overcome resistance or suspected encrustation and should not replace manufacturer instructions, passive deflation guidance, or urological assessment when removal is difficult.

Within this deliberately narrow research framework, the review highlights an understudied mechanical step and the need to document how the balloon was deflated. Incorporation into international or routine local guidelines remains premature. Comparative trials should include passive deflation as a clinically relevant comparator and establish safety before implementation.

These findings are hypothesis-generating and support further comparative research; they do not justify immediate integration into evidence-based nursing pathways.

### 4.5. Limitations

Several limitations warrant emphasis. All 46 trials were conducted in China, and no eligible international or cross-national multicentre trial was identified. Differences in catheter material, diameter, balloon design, deflation practice, dwell time, sex distribution, prostatic hyperplasia status, specialty, and perioperative care limit transferability. Most studies were small, single-centre, and not prospectively registered; predictable allocation was common, allocation concealment was reported poorly, operator blinding was infeasible, and blinded outcome assessment was uncommon. Pain instruments and reconstructed data formats varied, resulting in substantial heterogeneity and measurement concerns. Hedges’ g reduces small-sample bias but cannot resolve conceptual differences among pain instruments. Sex-stratified outcomes, prostatic hyperplasia status, catheter diameter and material, dwell time, balloon geometry, and withdrawal force were reported too incompletely for adjusted analyses or meta-regression. Urinary retention, spontaneous voiding, and catheter reinsertion were defined inconsistently and are not specific markers of urethral trauma. Comparative data on infection and other serious adverse events were unavailable, so safety cannot be established. The archived search records did not preserve per-database counts, a reproducible Google Scholar depth, or a complete grey literature audit, and paired screening decisions were unavailable for retrospective agreement statistics. Supplementary File S6 provides study-level numerical inputs for the seven overall meta-analyses and reproduces the reported pooled results. However, complete analytic code and ordinal conversion flags were not retained, so the sensitivity analysis excluding reconstructed ordinal data cannot be reproduced independently. Funnel plot asymmetry suggests that small positive studies may overestimate benefit. These limitations underpin the low or very low certainty ratings.

### 4.6. Future Research

Priority questions are whether reinjection adds benefit to passive deflation, which medium and volume are optimal if benefit exists, and whether effects vary by catheter material, diameter, dwell time, sex, or clinical context. Future studies should be international, multicentre, prospectively registered randomized trials with concealed allocation and standardized catheter reporting. They should provide sex-stratified results when clinically justified, use blinded outcome assessment where feasible, and prespecify monitoring of pain, hematuria, retention, reinsertion, infection, difficult removal, and longer-term urethral outcomes.

Knowledge translation should therefore begin with research standardization: explicit comparator definitions, manufacturer-consistent passive deflation, complete reporting of catheter and baseline characteristics, and prospective adverse event surveillance. Routine pathway adoption should await confirmatory trials in diverse settings.

## 5. Conclusions

Small-volume post-aspiration balloon reinjection was associated with less pain, hematuria, urinary retention, and catheter reinsertion and with more successful spontaneous voiding in 46 Chinese randomized or quasi-randomized trials. These associations compare reinjection with active complete aspiration and cannot be extrapolated to passive deflation protocols. Certainty was low or very low because of risk of bias, heterogeneity, incomplete reporting of catheter and baseline characteristics, single-country evidence, and suspected small-study effects. No extractable comparative data on infection or other serious adverse events were available; safety therefore remains uncertain.

The evidence does not justify routine implementation or a claim of established safety. Carefully governed comparative research may be reasonable in uncomplicated catheter removal, but international multicentre randomized trials that include passive deflation as a comparator are needed before wider adoption.

The principal implication is a research agenda focused on catheter withdrawal mechanics and the transferability of geographically concentrated procedural evidence, rather than an actionable treatment recommendation.

## Figures and Tables

**Table 1:** Abbreviated characteristics of selected included studies.

Study	Sample Control/Intervention	Population	Post-Aspiration Reinjection	Medium	Pain Assessment	Outcomes
Chen JQ 2021 [35]	98/98	Trauma orthopedics patients with preoperative catheterization	0.3–0.5 mL air reinjection	air	Other/unclear	urinary retention; catheter reinsertion
Chen L 2020 [36]	192/226	Male patients after flexible ureteroscopy	0.5 mL sterile water reinjection	liquid	numeric rating scale; facial pain scale	pain
Chen S 2025 [37] (0.3/0.5 mL)	30/60 *	Cerebral-infarction intervention; short-term catheterization	0.3 or 0.5 mL sterile water	liquid	visual analogue scale	pain; hematuria
Cao H 2013 [38]	60/60	Orthopedic surgery patients with indwelling catheters	0.5–1.0 mL saline retained/reinjected	liquid	numeric rating scale; verbal rating or ordinal scale	hematuria; urinary retention
Chen HL 2010 [39]	30/30	Male patients with balloon urinary catheters	0.5–1.0 mL saline reinjection	liquid	Not reported	hematuria
Dong YR 2017 [40]	62/62	Surgical patients with indwelling urinary catheters	Small-volume fluid reinjection	liquid	visual analogue scale; verbal rating or ordinal scale	pain; successful spontaneous voiding
Fan YY 2010 [41]	50/50	Male patients after thoracic surgery	0.5 mL saline reinjection	liquid	Other/unclear	successful spontaneous voiding
Gao YX 2013 [42]	50/50	Patients with indwelling balloon catheters	1.0 mL fluid reinjection	liquid	verbal rating or ordinal scale	hematuria; successful spontaneous voiding
Guan WL 2013 [43]	43/43	Patients with postoperative urinary retention and balloon catheters	0.5 mL air reinjection	air	Not reported	hematuria; urinary retention
Huang JM 2013 [44]	94/94	Urological endoscopic surgery patients	0.3–0.4 mL saline reinjection	liquid	numeric rating scale; verbal rating or ordinal scale	pain
Li FD 2013 [45]	100/100	Male catheterized patients	0.2–0.8 mL sterile saline, adjusted by catheter size and dwell time	liquid	Other/unclear	pain; hematuria; urinary retention; catheter reinsertion
Li Y 2015 [46]	50/50	Patients after craniocerebral tumor resection	0.5 mL saline reinjection	liquid	Not reported	successful spontaneous voiding; catheter reinsertion

The full 46-study version of Table 1 is provided in Supplementary File S2; the abbreviated in-text table shows the first 12 studies in chronological extraction order to comply with journal table limits. * For Chen S 2025 [37], 30/60 denotes one shared control group (n = 30) and two reinjection arms combined for pain (n = 60; n = 30 per volume); the control was counted once.

**Table 2:** Summary of findings and certainty of evidence.

Outcome	Studies	Participants	Effect Estimate	Heterogeneity	Certainty of Evidence	Main Reasons for Downgrading	Clinical Interpretation
Pain intensity during catheter removal	26	3900	SMD −1.14 (−1.35 to −0.92)	I^2^ = 89.8%	Very low	Serious risk of bias; very serious inconsistency; serious indirectness from single-country evidence and incompletely reported sex/catheter characteristics; suspected small-study effects	Lower pain; magnitude uncertain.
Urinary retention	16	2206	RR 0.22 (0.14 to 0.32)	I^2^ = 0.0%	Low	Serious risk of bias; serious indirectness from single-country evidence and incomplete baseline/catheter reporting	Lower retention; baseline risk matters.
Successful spontaneous voiding	19	2460	RR 1.29 (1.21 to 1.39)	I^2^ = 65.8%	Very low	Serious risk of bias; inconsistency; serious indirectness; suspected small-study effects	Higher voiding; definitions varied.
Any hematuria	20	2511	RR 0.21 (0.14 to 0.32)	I^2^ = 52.5%	Very low	Serious risk of bias; inconsistency; serious indirectness; suspected small-study effects	Lower hematuria; definitions varied.
Macroscopic hematuria	14	1863	RR 0.28 (0.19 to 0.41)	I^2^ = 21.8%	Low	Serious risk of bias; serious indirectness; suspected small-study effects	Lower macroscopic hematuria; low certainty.
Microscopic hematuria	9	1055	RR 0.34 (0.22 to 0.52)	I^2^ = 78.8%	Very low	Serious risk of bias; serious inconsistency; serious indirectness	Lower microscopic hematuria; nine studies and high heterogeneity.
Catheter reinsertion	11	1620	RR 0.44 (0.28 to 0.69)	I^2^ = 0.0%	Low	Serious risk of bias; serious indirectness from single-country evidence and incomplete baseline/catheter reporting	Lower reinsertion; context and baseline risk matter.

SMD, standardized mean difference; RR, risk ratio; I^2^, proportion of variability attributable to heterogeneity. GRADE downgrading domains were risk of bias (limitations in study conduct), inconsistency (unexplained variation across studies), indirectness (limited applicability to the review question), imprecision (uncertainty around the effect), and publication bias (selective availability of results).

## Data Availability

No new data were created or analyzed in this study. Data sharing is not applicable to this article.

## Supplementary Materials

The following supporting information can be downloaded at https://www.mdpi.com/article/10.3390/healthcare14182974/s1, Supplementary File S1 (Supplementary_S1_Search_Strategies.docx) contains database-specific search strings, platforms, fields, dates, limits, and a traceability statement regarding unavailable historical counts. Supplementary File S2 (Supplementary_S2_Study_Characteristics_Table.xlsx) contains the full 46-study characteristics and an updated ReadMe describing the available fields, Chen S 2025 [37] multi-arm handling, and the relationship to the numerical dataset. Supplementary File S3 (Supplementary_S3_Secondary_Forest_Plots.docx) contains forest plots for secondary outcomes. Supplementary File S4 (Supplementary_S4_Funnel_Plots.docx) contains funnel plots only for outcomes with at least 10 studies; microscopic hematuria (nine studies) is excluded. Supplementary File S5 (Supplementary_S5_PRISMA_2020_Checklist.docx) contains the completed PRISMA 2020 checklist. Supplementary File S6 (Supplementary_S6_Study-Level_Outcome_Data.xlsx) contains study-level group means, standard deviations, event counts, and sample sizes for the seven pooled outcomes.
