# Supplementary material for: Post-Aspiration Balloon Reinjection During Urethral Catheter Removal in Adult Men and Women: A Systematic Review and Meta-Analysis of Randomized and Quasi-Randomized Trials
Source: Healthcare (Basel). 2026 Sep 11;14(18):2974. doi: 10.3390/healthcare14182974 (PMC13606671; doi:10.3390/healthcare14182974)
Supplement: Supplementary file 1 [file healthcare-14-02974-s001.zip › Supplementary_S1_Search_Strategies.pdf]

Supplementary File S1. Database-Specific Search Strategies

Post-aspiration balloon reinjection during urethral catheter removal

**Coverage**	Database inception to 9 July 2026
**Sources**	PubMed/MEDLINE; Embase; Cochrane Library; Google Scholar; CNKI; Wanfang Data; VIP; SinoMed
**Language limits**	None
**Study-design focus**	Randomized and quasi-randomized controlled trials; high-sensitivity terminology retained

Traceability note. The supplied archive contained the concept-level draft, the merged total (4,767 database records), and the final study set, but not the original line-by-line histories or per-database exports. The strategies below are executable database-specific reconstructions prepared from the archived concepts and manuscript. They are not claimed to be verbatim contemporaneous logs. Per-database counts are reported as NR (not recoverable) rather than invented. Initial searching and preliminary analyses preceded PROSPERO registration on 15 July 2026. Supplementary surveillance continued during manuscript preparation, but complete rerun logs were not archived; therefore, 9 July 2026 remains the reproducible formal search date. A present-day rerun would not recreate the 9 July 2026 counts because live databases continue to change.

Search overview and retained counts

**Database records**	4,767 (aggregate retained count; per-database allocation NR)
**Manual/reference-list records**	7
**Total identified**	4,774
**Duplicate/near-duplicate records**	4,580

**Other pre-screening removals**	61
**Records screened**	133
**Full texts assessed**	86
**Included trials**	46

1. PubMed/MEDLINE

**Platform**	PubMed.gov
**Search date**	9 July 2026
**Coverage**	Inception to 9 July 2026
**Limits**	No language or sex limit; humans not applied as a hard filter
**Retrieved**	NR from supplied archive

Search line 1 - catheter concept

```
("Urinary Catheters"[Mesh] OR "urinary catheter"[tiab] OR "urinary catheters"[tiab] OR "urethral catheter"[tiab] OR "urethral catheters"[tiab] OR "indwelling catheter"[tiab] OR "indwelling catheters"[tiab] OR "Foley catheter"[tiab] OR "Foley catheters"[tiab] OR catheterisation[tiab] OR catheterization[tiab])
```

Search line 2 - removal concept

```
(remov*[tiab] OR withdraw*[tiab] OR extubat*[tiab] OR decatheter*[tiab])
```

Search line 3 - reinjection/balloon concept

```
(reinject*[tiab] OR re-inject*[tiab] OR reinflat*[tiab] OR re-inflat*[tiab] OR reinstill*[tiab]
OR re-instill*[tiab] OR "balloon cuffing"[tiab] OR "cuffing effect"[tiab] OR ((balloon[tiab] OR
cuff[tiab]) AND (deflat*[tiab] OR aspirat*[tiab] OR fold*[tiab] OR ridge*[tiab])))
```

Search line 4 - controlled-trial concept

```
(randomized controlled trial[pt] OR controlled clinical trial[pt] OR random*[tiab] OR trial[tiab]
OR quasi-random*[tiab] OR quasirandom*[tiab] OR control*[tiab] OR allocat*[tiab])
```

Final PubMed query

```
#1 AND #2 AND #3 AND #4
```

2. Embase

**Platform**	Embase.com (Elsevier)
**Search date**	9 July 2026
**Coverage**	Inception to 9 July 2026
**Fields**	Emtree; title, abstract, author keywords
**Limits**	None
**Retrieved**	NR from supplied archive

Search line 1 - catheter concept

```
'urinary catheter'/exp OR 'foley catheter':ti,ab,kw OR 'urinary catheter':ti,ab,kw OR 'urethral
catheter':ti,ab,kw OR 'indwelling catheter':ti,ab,kw
```

Search line 2 - removal concept

```
'catheter removal'/exp OR remov*:ti,ab,kw OR withdraw*:ti,ab,kw OR extubat*:ti,ab,kw OR
decatheter*:ti,ab,kw
```

Search line 3 - reinjection/balloon concept

```
reinject*:ti,ab,kw OR 're-inject*':ti,ab,kw OR reinflat*:ti,ab,kw OR 're-inflat*':ti,ab,kw OR
reinstill*:ti,ab,kw OR 'balloon cuffing':ti,ab,kw OR 'cuffing effect':ti,ab,kw OR ((balloon OR
cuff):ti,ab,kw AND (deflat* OR aspirat* OR fold* OR ridge*):ti,ab,kw)
```

Search line 4 - controlled-trial concept

```
'randomized controlled trial'/exp OR 'controlled clinical trial'/exp OR random*:ti,ab,kw OR
trial:ti,ab,kw OR 'quasi random*':ti,ab,kw OR control*:ti,ab,kw OR allocat*:ti,ab,kw
```

Final Embase query

```
#1 AND #2 AND #3 AND #4
```

3. Cochrane Library

**Platform**	Cochrane Library - Search Manager
**Search date**	9 July 2026
**Coverage**	Inception to 9 July 2026
**Fields**	MeSH; title, abstract, keyword
**Limits**	None
**Retrieved**	NR from supplied archive

Search line 1

```
MeSH descriptor: [Urinary Catheters] explode all trees
```

Search line 2

```
((Foley NEXT catheter*) OR (urinary NEXT catheter*) OR (urethral NEXT catheter*) OR (indwelling
NEXT catheter*)):ti,ab,kw
```

Search line 3

```
(remov* OR withdraw* OR extubat* OR decatheter*):ti,ab,kw
```

Search line 4

```
(reinject* OR re-inject* OR reinflat* OR re-inflat* OR reinstill* OR "balloon cuffing" OR
"cuffing effect" OR ((balloon OR cuff) NEAR/3 (deflat* OR aspirat* OR fold* OR ridge*))):ti,ab,kw
```

Final Cochrane query

```
(#1 OR #2) AND #3 AND #4
```

4. Google Scholar (supplementary search)

**Platform**	Google Scholar
**Search date**	9 July 2026
**Role**	Supplementary citation discovery; not treated as a comprehensive database search
**Result-depth limit**	NR - archived log did not preserve the ranked-result cutoff or export depth
**Retrieved**	NR from supplied archive

Query A

```
"Foley catheter" removal reinjection balloon pain trial
```

Query B

```
"urinary catheter" removal "balloon reinjection"
```

Query C

```
"urethral catheter" withdrawal reinflation cuffing
```

Query D

```
"catheter balloon cuffing" removal
```

Procedure

```
Run each query separately; screen the ranked results and reference lists. Because the original
screening depth was not retained, no retrospective cutoff or count is asserted.
```

5. China National Knowledge Infrastructure (CNKI)

**Platform**	CNKI - China Academic Journals / Professional Search
**Search date**	9 July 2026
**Coverage**	Inception to 9 July 2026
**Field**	SU = subject (title, keywords, abstract)
**Limits**	None
**Retrieved**	NR from supplied archive

Professional-search string (Chinese, as executed):

```
SU=('留置导尿管'+'气囊导尿管'+'球囊导尿管'+'Foley 导尿管'+'尿管') * SU=('拔除'+'拔管'+'尿管拔除'+'导尿
管拔除') * SU=('回注'+'再注入'+'重新注入'+'再充盈'+'保留少量液体'+'保留少量气体'+'气囊回注'+'球囊回注
'+'袖带效应'+'皱褶')
```

English translation:

*SU = ('indwelling urinary catheter' OR 'balloon catheter' OR 'Foley catheter' OR 'urinary catheter') * SU = ('removal' OR 'extubation' OR 'urinary catheter removal') * SU = ('reinjection' OR 're-injection' OR*

‘reinflation’ OR ‘re-inflation’ OR ‘small-volume fluid retention’ OR ‘small-volume gas retention’ OR ‘balloon reinjection’ OR ‘cuffing effect OR folds’)

Sensitivity string

```
SU=('导尿管'+'尿管') * SU=('拔除'+'拔管') * SU=('疼痛'+'血尿'+'尿潴留'+'自主排尿'+'重新插管') * SU=('随机'+'对照'+'临床试验')
```

English translation:
*SU=(‘urinary catheter’ OR ‘catheter’ * SU=(‘removal’ OR ‘extubation’) * SU=(‘pain’ OR ‘hematuria’ OR ‘urinary retention’ OR ‘spontaneous voiding’ OR ‘catheter reinsertion’) * SU=(‘randomized’ OR ‘controlled’ OR ‘clinical trial’)*

6. Wanfang Data

**Platform**	Wanfang Data Knowledge Service
**Search date**	9 July 2026
**Coverage**	Inception to 9 July 2026
**Field**	Topic
**Limits**	None
**Retrieved**	NR from supplied archive

Advanced-search string

```
主题:(留置导尿管 OR 气囊导尿管 OR 球囊导尿管 OR Foley导尿管 OR 尿管) AND 主题:(拔除 OR 拔管 OR 尿管拔除 OR 导尿管拔除) AND 主题:(回注 OR 再注入 OR 重新注入 OR 再充盈 OR 保留少量液体 OR 保留少量气体 OR 气囊回注 OR 球囊回注 OR 袖带效应 OR 皱褶)
```

English translation:
Topic : (‘indwelling urinary catheter’ OR ‘balloon catheter’ OR ‘Foley catheter’ OR ‘urinary catheter’) AND (‘removal’ OR ‘extubation’ OR ‘urinary catheter removal’) AND (‘reinjection’ OR ‘re-injection’ OR ‘reinflation’ OR ‘re-inflation’ OR ‘small-volume fluid retention’ OR ‘small-volume gas retention’ OR ‘balloon reinjection’ OR ‘cuffing effect OR folds’)

Sensitivity string

```
主题:(导尿管 OR 尿管) AND 主题:(拔除 OR 拔管) AND 主题:(疼痛 OR 血尿 OR 尿潴留 OR 自主排尿 OR 重新插管) AND 主题:(随机 OR 对照 OR 临床试验)
```

English translation:
Topic : ('urinary catheter' OR 'catheter') AND ('removal' OR 'extubation') AND ('pain' OR 'hematuria' OR 'urinary retention' OR 'spontaneous voiding' OR 'catheter reinsertion') AND ('randomized' OR 'controlled' OR 'clinical trial')

7. VIP Chinese Science and Technology Journals Database

**Platform**	VIP / CQVIP
**Search date**	9 July 2026
**Coverage**	Inception to 9 July 2026
**Field**	M = title/keyword/abstract
**Limits**	None
**Retrieved**	NR from supplied archive

Professional-search string

```
M=(留置导尿管 OR 气囊导尿管 OR 球囊导尿管 OR Foley 导尿管 OR 尿管) AND M=(拔除 OR 拔管 OR 尿管拔除 OR 导尿管拔除) AND M=(回注 OR 再注入 OR 重新注入 OR 再充盈 OR 保留少量液体 OR 保留少量气体 OR 气囊回注 OR 球囊回注 OR 袖带效应 OR 皱褶)
```

English translation:
M = ('indwelling urinary catheter' OR 'balloon catheter' OR 'Foley catheter' OR 'urinary catheter') AND M = ('removal' OR 'extubation' OR 'urinary catheter removal') AND M = ('reinjection' OR 're-injection' OR 'reinflation' OR 're-inflation' OR 'small-volume fluid retention' OR 'small-volume gas retention' OR 'balloon reinjection' OR 'cuffing effect OR folds')

Sensitivity string

```
M=(导尿管 OR 尿管) AND M=(拔除 OR 拔管) AND M=(疼痛 OR 血尿 OR 尿潴留 OR 自主排尿 OR 重新插管) AND M=(随机 OR 对照 OR 临床试验)
```

English translation:
M=('urinary catheter' OR 'catheter' AND M=('removal' OR 'extubation') AND M=('pain' OR 'hematuria' OR 'urinary retention' OR 'spontaneous voiding' OR 'catheter reinsertion') AND M=('randomized' OR 'controlled' OR 'clinical trial')

8. SinoMed

**Platform**	SinoMed / Chinese Biomedical Literature Service System
**Search date**	9 July 2026
**Coverage**	Inception to 9 July 2026
**Field**	Common fields (title, abstract, keywords, subject terms)
**Limits**	None
**Retrieved**	NR from supplied archive

Advanced-search string

```
(留置导尿管 OR 气囊导尿管 OR 球囊导尿管 OR Foley 导尿管 OR 尿管)/常用字段 AND (拔除 OR 拔管 OR 尿管拔除 OR 导尿管拔除)/常用字段 AND (回注 OR 再注入 OR 重新注入 OR 再充盈 OR 保留少量液体 OR 保留少量气体 OR 气囊回注 OR 球囊回注 OR 袖带效应 OR 皱褶)/常用字段
```

English translation:
('indwelling urinary catheter' OR 'balloon catheter' OR 'Foley catheter' OR 'urinary catheter') / Common fields AND ('removal' OR 'extubation' OR 'urinary catheter removal') / Common fields AND ('reinjection' OR 're-injection' OR 'reinflation' OR 're-inflation' OR 'small-volume fluid retention' OR 'small-volume gas retention' OR 'balloon reinjection' OR 'cuffing effect OR folds') / Common fields

Sensitivity string

```
(导尿管 OR 尿管)/常用字段 AND (拔除 OR 拔管)/常用字段 AND (疼痛 OR 血尿 OR 尿潴留 OR 自主排尿 OR 重新插管)/常用字段 AND (随机 OR 对照 OR 临床试验)/常用字段
```

English translation:
(‘urinary catheter’ OR ‘catheter’) / Common fields AND (‘removal’ OR ‘extubation’) / Common fields AND (‘pain’ OR ‘hematuria’ OR ‘urinary retention’ OR ‘spontaneous voiding’ OR ‘catheter reinsertion’) / Common fields AND (‘randomized’ OR ‘controlled’ OR ‘clinical trial’) / Common fields

Deduplication and supplementary checking

All database exports were merged. Exact identifiers (PMID, DOI, and database accession number) were used first. Remaining records were matched on normalized title, first author, and publication year, followed by manual review of near-duplicate titles. Reference lists of included reports and relevant reviews were hand-searched. The supplied archive retained aggregate counts only; database-specific yields and the Google Scholar result-depth cutoff were not retrospectively reconstructed. Supplementary surveillance for potentially higher-quality reports continued during manuscript preparation, but was not treated as a reproducible database update because complete rerun logs were unavailable.
