# Supplementary material for: Post-Aspiration Balloon Reinjection During Urethral Catheter Removal in Adult Men and Women: A Systematic Review and Meta-Analysis of Randomized and Quasi-Randomized Trials
Source: Healthcare (Basel). 2026 Sep 11;14(18):2974. doi: 10.3390/healthcare14182974 (PMC13606671; doi:10.3390/healthcare14182974)

Supplementary File S3. Secondary-outcome forest plots. Each random-effects plot reports the pooled effect with 95% CI, I², τ², and Cochran Q.

Abbreviations: RR, risk ratio; CI, confidence interval; I², proportion of variability attributable to heterogeneity; τ², between-study variance; Q, Cochran heterogeneity statistic.

Urinary retention

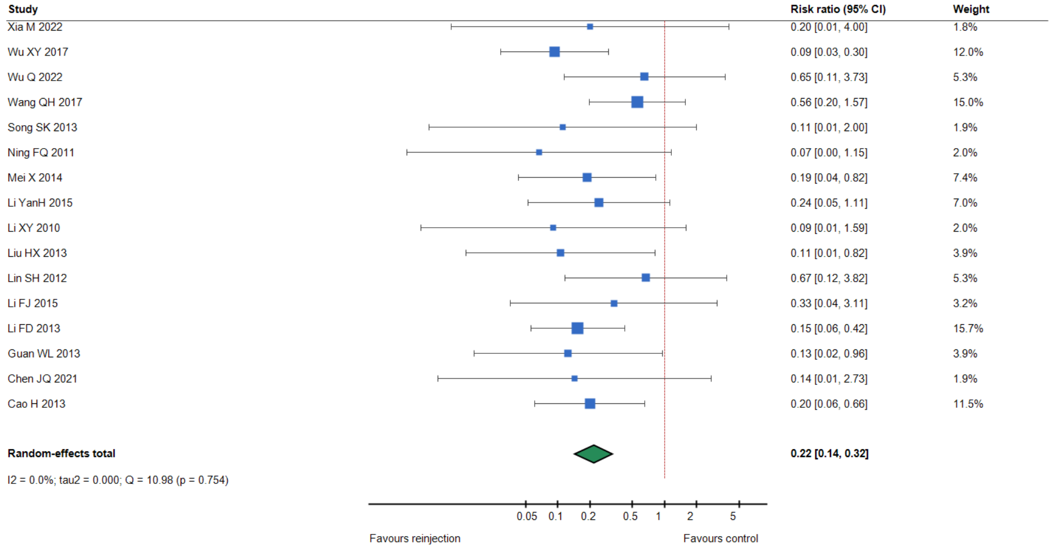

Successful spontaneous voiding

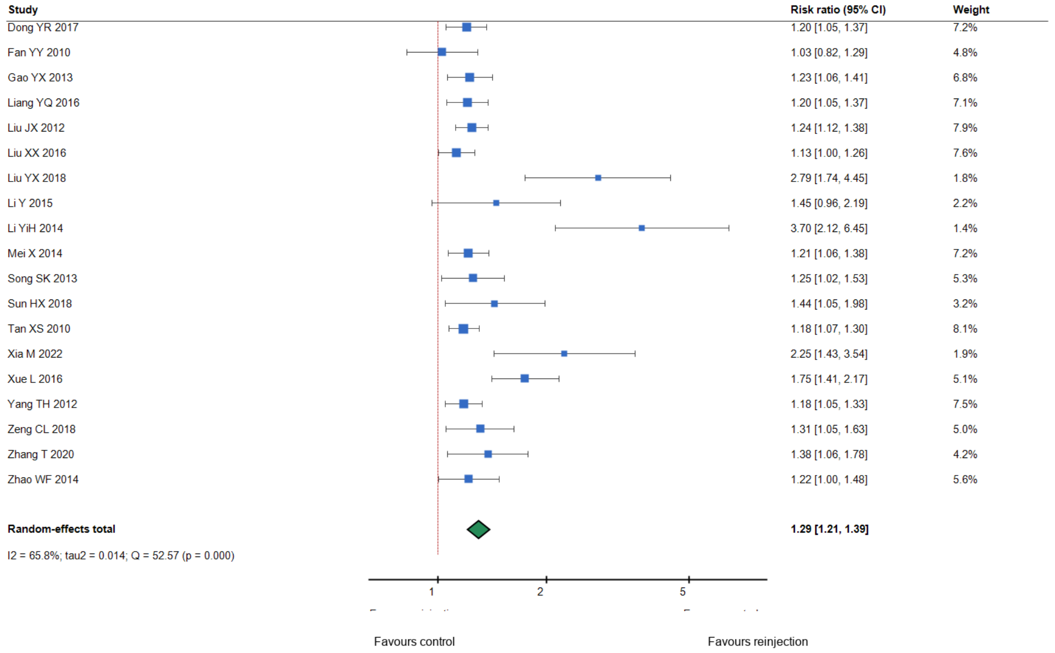

Any hematuria

Study	Risk ratio (95% CI)	Weight
Cao H 2013	0.20 [0.02, 1.66]	2.9%
Chen HL 2010	0.17 [0.07, 0.44]	7.8%
Chen S 2025	0.17 [0.02, 1.30]	3.0%
Gao YX 2013	0.08 [0.00, 1.33]	1.8%
Guan WL 2013	0.45 [0.28, 0.72]	11.3%
Liang YQ 2016	0.05 [0.00, 0.80]	1.8%
Li FD 2013	0.31 [0.12, 0.82]	7.6%
Li FJ 2015	0.13 [0.02, 0.97]	3.1%
Lin SH 2012	0.50 [0.05, 5.34]	2.4%
Liu HX 2013	0.32 [0.18, 0.55]	10.7%
Liu YX 2018	0.13 [0.02, 0.96]	3.1%
Li YanH 2015	0.64 [0.39, 1.03]	11.2%
Li YuH 2011	0.02 [0.00, 0.13]	3.3%
Mei X 2014	0.21 [0.02, 1.74]	2.9%
Ning FQ 2011	0.04 [0.01, 0.14]	5.3%
Ruan Q 2010	0.11 [0.01, 0.85]	3.1%
Song SK 2013	0.15 [0.04, 0.64]	5.1%
Sun WL 2017	0.36 [0.17, 0.78]	9.0%
Wang QH 2017	0.20 [0.02, 1.67]	2.9%
Wang SY 2012	0.02 [0.00, 0.39]	1.9%
**Random-effects total**	**0.21 [0.14, 0.32]**	

I2 = 52.5%; tau2 = 0.328; Q = 40.03 (p = 0.003)

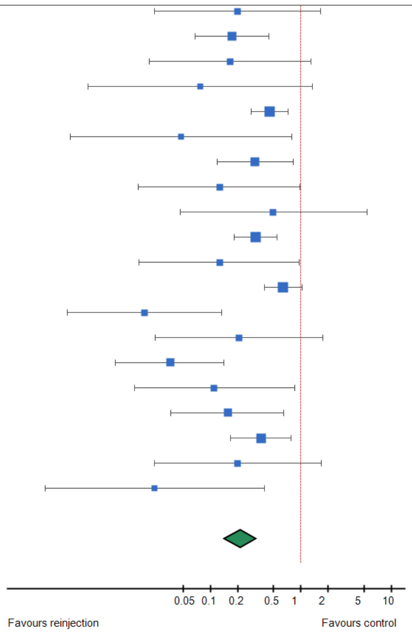

Macroscopic hematuria

Study	Risk ratio (95% CI)	Weight
Cao H 2013	0.20 [0.02, 1.66]	2.9%
Chen HL 2010	0.17 [0.07, 0.44]	11.4%
Gao YX 2013	0.08 [0.00, 1.33]	1.7%
Liang YQ 2016	0.05 [0.00, 0.80]	1.7%
Li FD 2013	0.31 [0.12, 0.82]	10.8%
Li FJ 2015	0.13 [0.02, 0.97]	3.1%
Lin SH 2012	0.50 [0.05, 5.34]	2.4%
Liu YX 2018	0.13 [0.02, 0.96]	3.1%
Li YanH 2015	0.64 [0.39, 1.03]	23.3%
Mei X 2014	0.21 [0.02, 1.74]	2.9%
Ruan Q 2010	0.11 [0.01, 0.85]	3.1%
Sun WL 2017	0.36 [0.17, 0.78]	14.7%
Wu GY 2009	0.23 [0.11, 0.47]	15.9%
Wu Q 2022	0.16 [0.02, 1.30]	3.0%
**Random-effects total**	**0.28 [0.19, 0.41]**	

I2 = 21.8%; tau2 = 0.098; Q = 16.63 (p = 0.217)

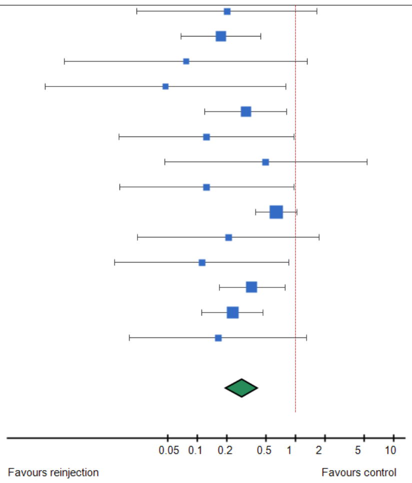

Microscopic hematuria

Study	Risk ratio (95% CI)	Weight
Guan WL 2013	0.45 [0.28, 0.72]	14.9%
Li FJ 2015	0.64 [0.49, 0.83]	17.0%
Liu HX 2013	0.32 [0.18, 0.55]	14.0%
Mei X 2014	0.29 [0.11, 0.74]	9.6%
Ning FQ 2011	0.04 [0.01, 0.14]	6.2%
Song SK 2013	0.15 [0.04, 0.64]	6.0%
Wang SY 2012	0.02 [0.00, 0.39]	2.1%
Wu Q 2022	0.73 [0.55, 0.98]	16.7%
Zhang JF 2003	0.31 [0.18, 0.55]	13.6%
**Random-effects total**	**0.34 [0.22, 0.52]**	

I2 = 78.8%; tau2 = 0.261; Q = 37.71 (p = 0.000)

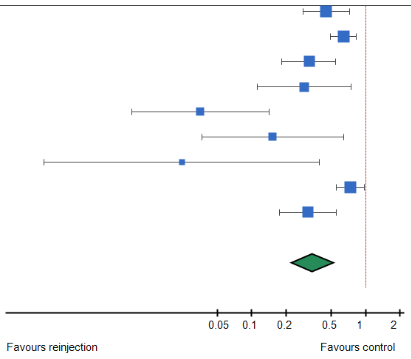

Catheter reinsertion

Study	Risk ratio (95% CI)	Weight
Chen JQ 2021	0.34 [0.01, 8.17]	2.0%
Li FD 2013	0.30 [0.09, 1.06]	12.7%
Li Y 2015	0.89 [0.37, 2.12]	26.7%
Li YanH 2015	0.14 [0.02, 1.13]	4.6%
Li YiH 2014	0.33 [0.04, 3.08]	4.1%
Sun HX 2018	0.22 [0.03, 1.80]	4.5%
Tan XS 2010	0.20 [0.01, 4.03]	2.2%
Xue L 2016	0.40 [0.16, 0.99]	24.3%
Zeng CL 2018	0.11 [0.01, 1.99]	2.4%
Zhang T 2020	0.20 [0.02, 1.66]	4.5%
Zhao WF 2014	0.80 [0.22, 2.91]	12.1%
**Random-effects total**	**0.44 [0.28, 0.69]**	

I2 = 0.0%; tau2 = 0.000; Q = 7.08 (p = 0.718)

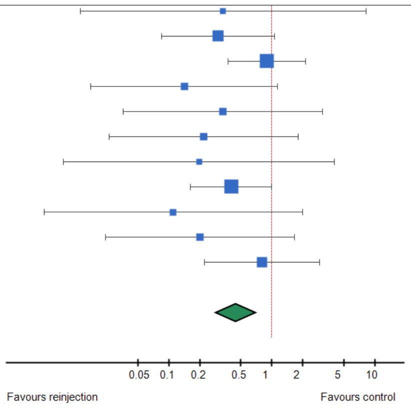

Supplement: Supplementary file 1 [file healthcare-14-02974-s001.zip › Supplementary_S3_Secondary_Forest_Plots.pdf]
