# Supplementary material for: Post-Aspiration Balloon Reinjection During Urethral Catheter Removal in Adult Men and Women: A Systematic Review and Meta-Analysis of Randomized and Quasi-Randomized Trials
Source: Healthcare (Basel). 2026 Sep 11;14(18):2974. doi: 10.3390/healthcare14182974 (PMC13606671; doi:10.3390/healthcare14182974)

Supplementary File S4. Funnel plots for outcomes with at least 10 studies. Microscopic hematuria (nine studies) is excluded.

Funnel plots display study effect estimates against standard error. SMD, standardized mean difference; RR analyses are plotted on the log-risk-ratio scale.

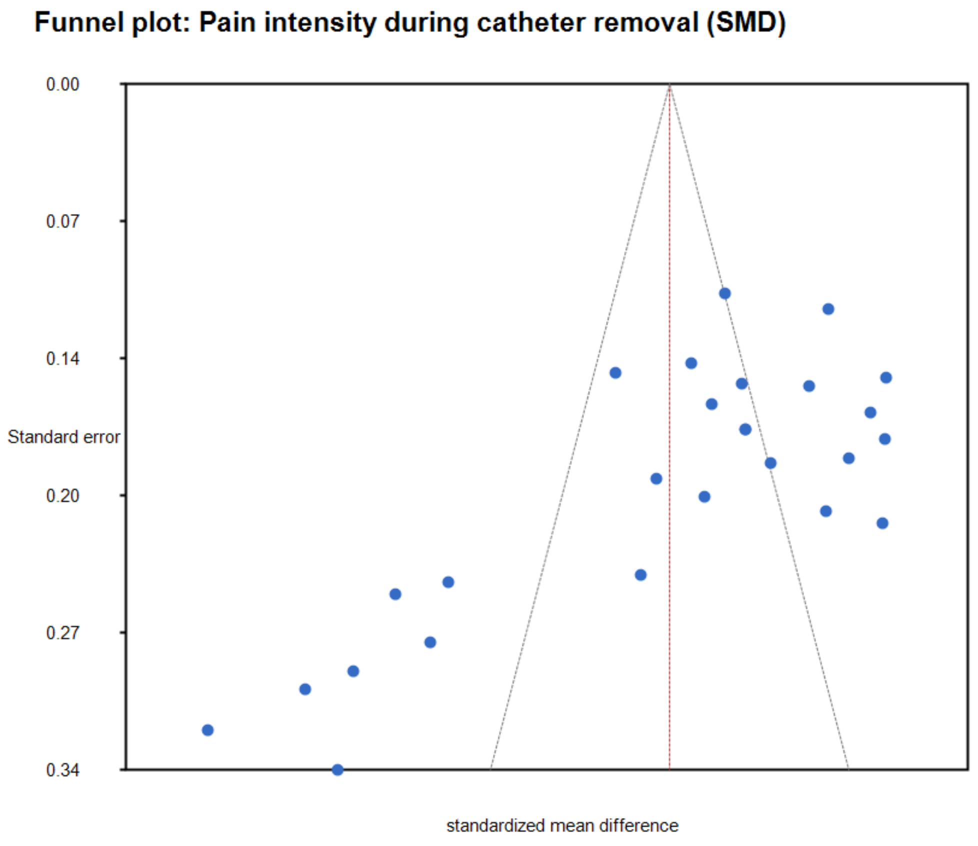

## Funnel plot: Any hematuria

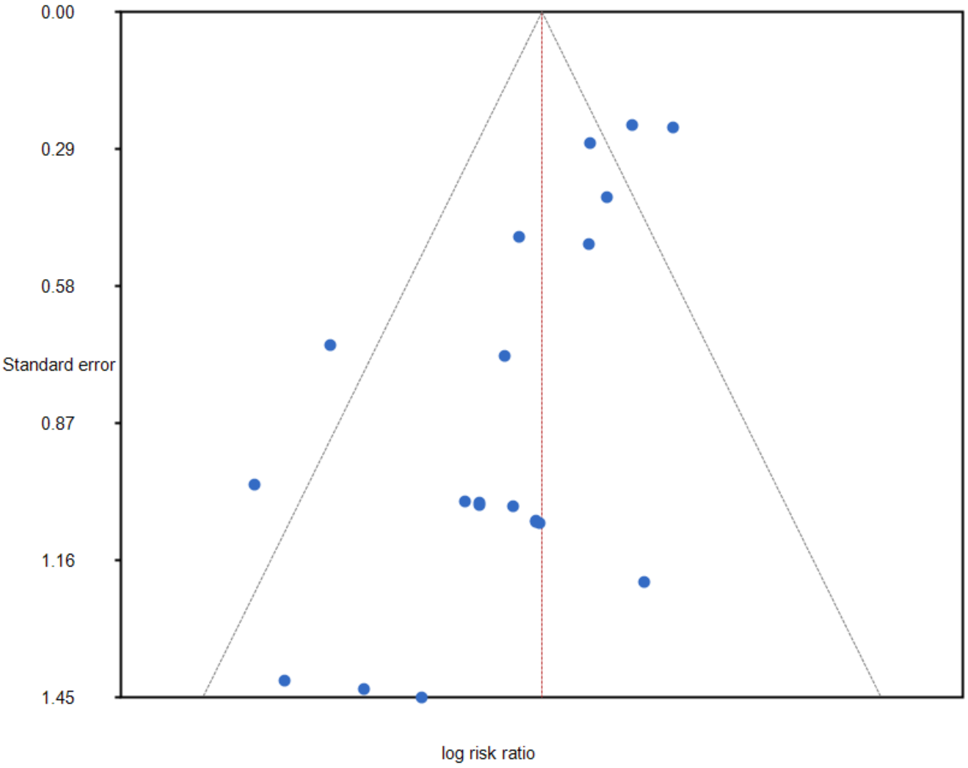

## Funnel plot: Macroscopic hematuria

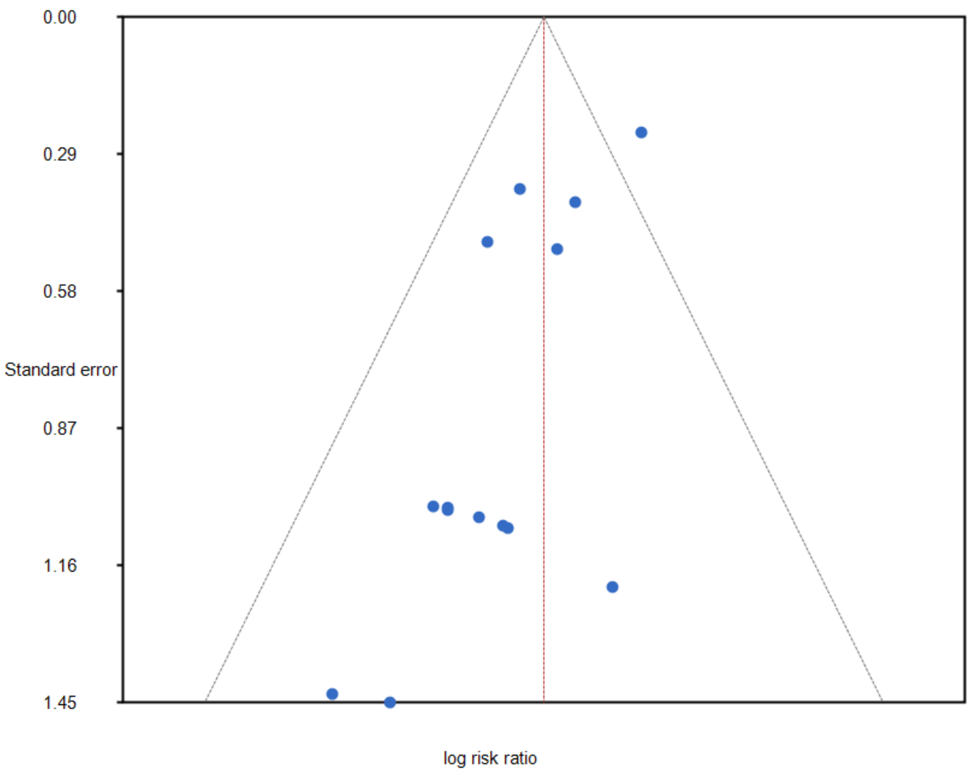

## Funnel plot: Catheter reinsertion

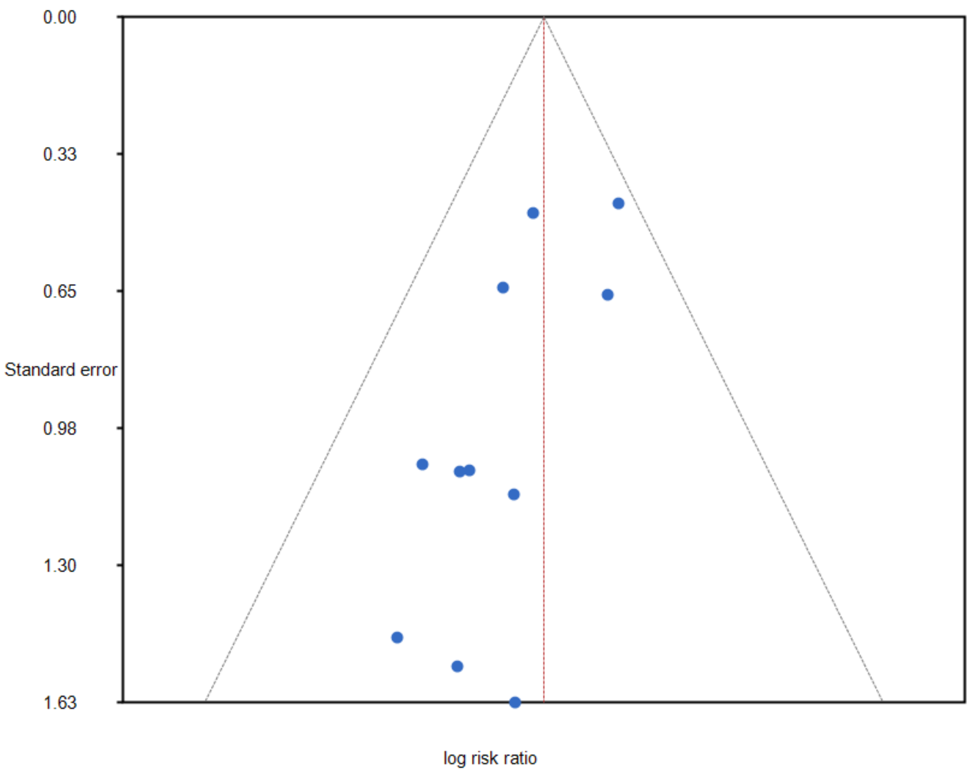

**Funnel plot: Successful spontaneous voiding**

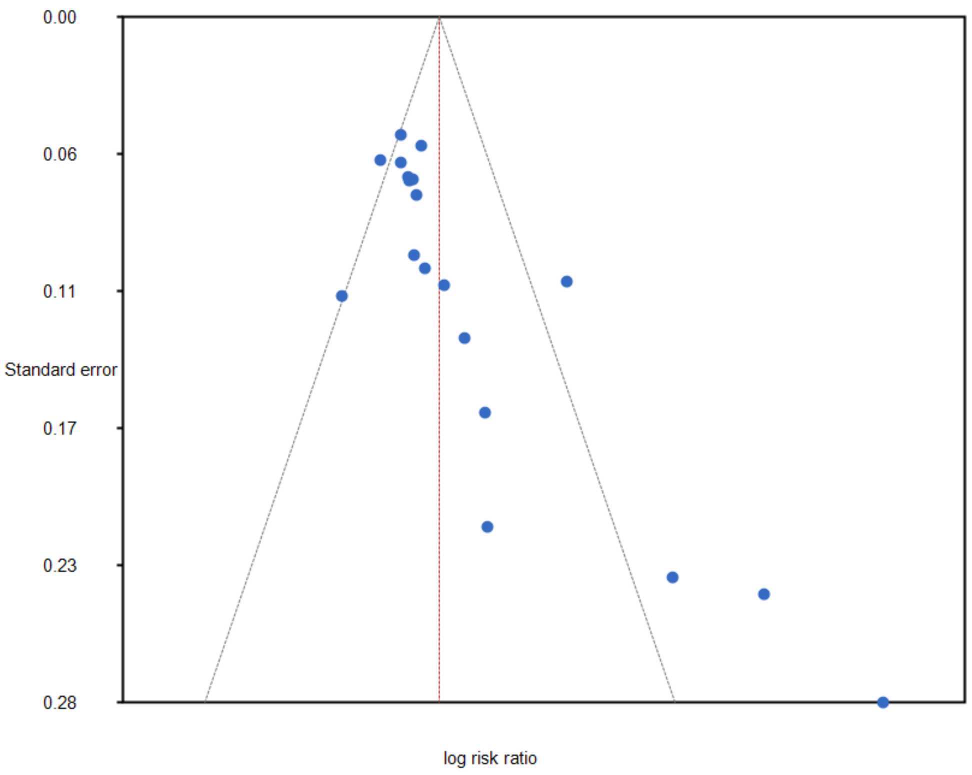

## Funnel plot: Urinary retention

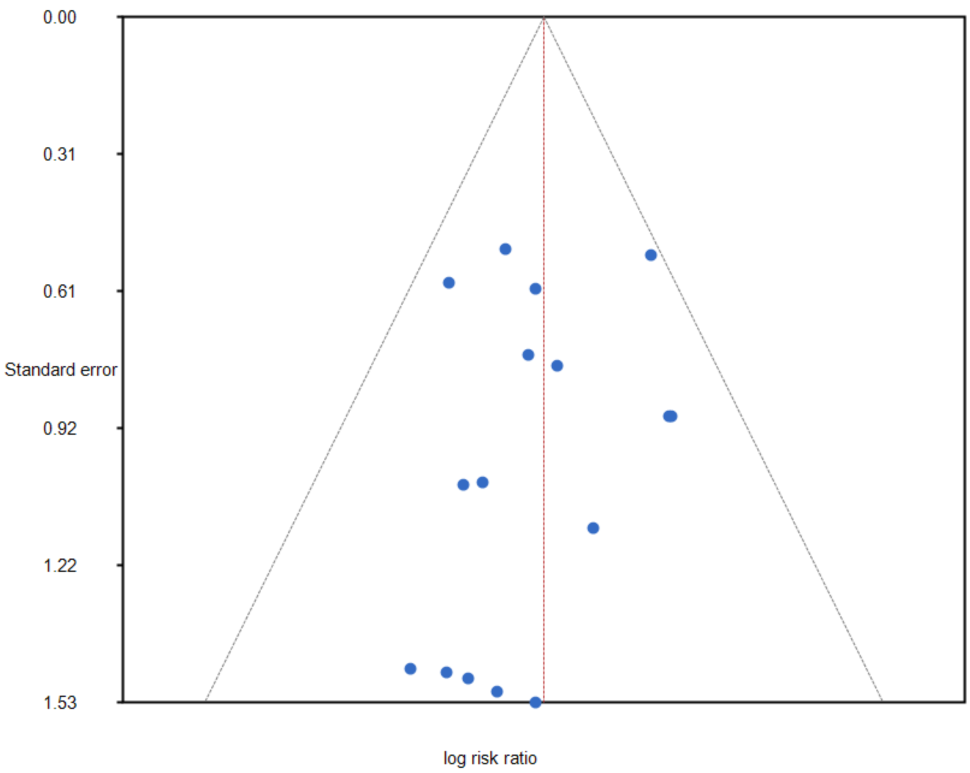

Supplement: Supplementary file 1 [file healthcare-14-02974-s001.zip › Supplementary_S4_Funnel_Plots.pdf]
