# Supplementary material for: Post-Aspiration Balloon Reinjection During Urethral Catheter Removal in Adult Men and Women: A Systematic Review and Meta-Analysis of Randomized and Quasi-Randomized Trials
Source: Healthcare (Basel). 2026 Sep 11;14(18):2974. doi: 10.3390/healthcare14182974 (PMC13606671; doi:10.3390/healthcare14182974)
Supplement: Supplementary file 1 [file healthcare-14-02974-s001.zip › Supplementary_S5_PRISMA_2020_Checklist_revised.pdf]

PRISMA 2020 Checklist

Section and Topic	Item #	Checklist item	Location where item is reported
**TITLE**			
Title	1	Identify the report as a systematic review.	Full title identifies a systematic review and meta-analysis (Manuscript p1).
**ABSTRACT**			
Abstract	2	See the PRISMA 2020 for Abstracts checklist.	Structured Abstract reports PICOS, pooled effects, certainty, registration chronology, adverse-event reporting limits, and applicability limits (Manuscript pp1-2).
**INTRODUCTION**			
Rationale	3	Describe the rationale for the review in the context of existing knowledge.	Section 1 explains guideline context, the cuffing mechanism, the geographically restricted evidence base, examples of geographic practice variation in cardiac surgery, and the unresolved clinical question (Manuscript pp. 2-3).
Objectives	4	Provide an explicit statement of the objective(s) or question(s) the review addresses.	Final Introduction paragraphs state the three objectives and the cautious research question (Manuscript p3).
**METHODS**			
Eligibility criteria	5	Specify the inclusion and exclusion criteria for the review and how studies were grouped for the syntheses.	Section 2.3 specifies population, intervention, comparator, outcomes, study design, and synthesis grouping (Manuscript pp4).
Information sources	6	Specify all databases, registers, websites, organisations, reference lists and other sources searched or consulted to identify studies. Specify the date when each source was last searched or consulted.	Section 2.2 lists all eight databases, manual checking, and the formal last-search date of 9 July 2026; supplementary surveillance is distinguished from a reproducible update (Manuscript p3-4; Supplementary S1).
Search strategy	7	Present the full search strategies for all databases, registers and websites, including any filters and limits used.	Supplementary File S1 (complete line-by-line MeSH/keyword search strings for each database)
Selection process	8	Specify the methods used to decide whether a study met the inclusion criteria of the review, including how many reviewers screened each record and each report retrieved, whether they worked independently, and if applicable, details of automation tools used in the process.	Section 2.6 reports dual independent screening, full-text assessment, and third-reviewer resolution; no automated

PRISMA

PRISMA 2020 Checklist

Section and Topic	Item #	Checklist item	Location where item is reported
			screening was used (Manuscript p5).
Data collection process	9	Specify the methods used to collect data from reports, including how many reviewers collected data from each report, whether they worked independently, any processes for obtaining or confirming data from study investigators, and if applicable, details of automation tools used in the process.	Section 2.6 reports dual independent extraction, the piloted form, and non-inference of missing data (Manuscript p5).
Data items	10a	List and define all outcomes for which data were sought. Specify whether all results that were compatible with each outcome domain in each study were sought (e.g. for all measures, time points, analyses), and if not, the methods used to decide which results to collect.	Section 2.5 defines the primary and secondary outcomes, hierarchy of pain measures, and timing (Manuscript pp5).
	10b	List and define all other variables for which data were sought (e.g. participant and intervention characteristics, funding sources). Describe any assumptions made about any missing or unclear information.	Sections 2.3-2.4 list study, participant, catheter, intervention, and design variables; missing values were recorded as not reported (Manuscript pp4; Supplementary S2).
Study risk of bias assessment	11	Specify the methods used to assess risk of bias in the included studies, including details of the tool(s) used, how many reviewers assessed each study and whether they worked independently, and if applicable, details of automation tools used in the process.	Section 2.7 reports Risk of Bias 2 domains, two independent assessors, and third-reviewer arbitration (Manuscript p5; Figure 2).
Effect measures	12	Specify for each outcome the effect measure(s) (e.g. risk ratio, mean difference) used in the synthesis or presentation of results.	Section 2.9 reports Hedges' g SMD and harmonized MD for pain and RR for binary outcomes (Manuscript p6).
Synthesis methods	13a	Describe the processes used to decide which studies were eligible for each synthesis (e.g. tabulating the study intervention characteristics and comparing against the planned groups for each synthesis (item #5)).	Eligible syntheses required the prespecified intervention/comparator; Chen S 2025 arms were combined without duplicate control counting (Manuscript p6).
	13b	Describe any methods required to prepare the data for presentation or synthesis, such as handling of missing summary statistics, or data conversions.	Section 2.5 describes ordinal-category conversion; missing numerical data were not imputed (Manuscript p5; reproducibility limitation pp12 and 13-14).
	13c	Describe any methods used to tabulate or visually display results of individual studies and syntheses.	Forest plots: Figure 3 and Supplementary S3; funnel plots: Supplementary S4; summary tables: Tables 1-2.
	13d	Describe any methods used to synthesize results and provide a rationale for the choice(s). If meta-analysis was performed, describe the model(s), method(s) to identify the presence and extent of statistical heterogeneity, and software package(s)	Random-effects inverse-variance/Mantel-Haenszel synthesis; I^2, τ^2, and Q; Review

PRISMA 2020 Checklist

Section and Topic	Item #	Checklist item	Location where item is reported
		used.	Manager (RevMan) version 5.3; 0.5 continuity correction for single-zero studies; double-zero studies excluded from that outcome (Section 2.9, Manuscript p6).
	13e	Describe any methods used to explore possible causes of heterogeneity among study results (e.g. subgroup analysis, meta-regression).	Prespecified effect modifiers included medium, volume, allocation method, sample size, specialty, sex, and catheter characteristics; incomplete reporting precluded meta-regression (Manuscript pp4 and 12).
	13f	Describe any sensitivity analyses conducted to assess robustness of the synthesized results.	High-risk-study exclusion, leave-one-out analysis, and fixed-effect reanalysis are reported; the ordinal-data exclusion analysis was not reproducible from the archive (Manuscript pp9-10 and 12).
Reporting bias assessment	14	Describe any methods used to assess risk of bias due to missing results in a synthesis (arising from reporting biases).	Funnel plots and Egger regression were restricted to outcomes with at least 10 studies; the change from the registry's three-study threshold is disclosed (Manuscript p6).
Certainty assessment	15	Describe any methods used to assess certainty (or confidence) in the body of evidence for an outcome.	Section 2.8 reports GRADE domains and reviewer workflow (Manuscript p6; Table 2).
**RESULTS**			
Study selection	16a	Describe the results of the search and selection process, from the number of records identified in the search to the number of studies included in the review, ideally using a flow diagram.	Section 3.1 and Figure 1 report the full selection flow (Manuscript pp6-7).
	16b	Cite studies that might appear to meet the inclusion criteria, but which were excluded, and explain why they were excluded.	Section 3.1 reports full-text exclusion categories and counts (Manuscript p6-7).
Study characteristics	17	Cite each included study and present its characteristics.	Table 1 provides an abbreviated summary; Supplementary S2 provides all 46 archived study-characteristic records (Manuscript p7-8).
Risk of bias in studies	18	Present assessments of risk of bias for each included study.	Figure 2 and Section 3.3 present study-level/domain risk-of-bias

PRISMA 2020 Checklist

Section and Topic	Item #	Checklist item	Location where item is reported
			judgments and aggregate counts (Manuscript p9).
Results of individual studies	19	For all outcomes, present, for each study: (a) summary statistics for each group (where appropriate) and (b) an effect estimate and its precision (e.g. confidence/credible interval), ideally using structured tables or plots.	Figure 3 reports individual pain effects; Supplementary S3 reports secondary-outcome study effects; Supplementary S6 provides the underlying study-level means/SDs or event counts and sample sizes. Zhang JF 2003 appears only in the microscopic-hematuria dataset because its full text did not measure successful spontaneous voiding (Manuscript pp9-10).
Results of syntheses	20a	For each synthesis, briefly summarise the characteristics and risk of bias among contributing studies.	Sections 3.3-3.5 summarize risk of bias and contributing-study context (Manuscript pp9-10-12).
	20b	Present results of all statistical syntheses conducted. If meta-analysis was done, present for each the summary estimate and its precision (e.g. confidence/credible interval) and measures of statistical heterogeneity. If comparing groups, describe the direction of the effect.	Table 2 and forest plots report pooled effects, 95% CIs, and heterogeneity statistics (Manuscript pp9-10-10-12; Supplementary S3).
	20c	Present results of all investigations of possible causes of heterogeneity among study results.	Section 3.6 reports descriptive subgroup comparisons and explains why adjusted meta-regression was not performed (Manuscript p12).
	20d	Present results of all sensitivity analyses conducted to assess the robustness of the synthesized results.	Section 3.6 reports high-risk exclusion, leave-one-out, and fixed-effect results and identifies the nonreproducible ordinal-data analysis (Manuscript p12).
Reporting biases	21	Present assessments of risk of bias due to missing results (arising from reporting biases) for each synthesis assessed.	Section 3.6 reports funnel asymmetry and Egger p<0.001 for pain; Supplementary S4 contains eligible funnel plots (Manuscript p12).
Certainty of evidence	22	Present assessments of certainty (or confidence) in the body of evidence for each outcome assessed.	Table 2 reports GRADE ratings and downgrade reasons for every outcome (Manuscript p10-12).
**DISCUSSION**			
Discussion	23a	Provide a general interpretation of the results in the context of other evidence.	Sections 4.1-4.3 interpret the findings against EAUN guidance, explain the geographic concentration of the evidence,

PRISMA 2020 Checklist

Section and Topic	Item #	Checklist item	Location where item is reported
			compare it with documented international variation in cardiac-surgery practice, and define comparator boundaries (Manuscript pp13-14).
	23b	Discuss any limitations of the evidence included in the review.	Section 4.5 discusses limitations of the included evidence, including single-country evidence and limits to transferability (Manuscript p14).
	23c	Discuss any limitations of the review processes used.	Section 4.5 discusses search-log, screening-record, numerical-input, code, and sensitivity-analysis limitations (Manuscript p13-14).
	23d	Discuss implications of the results for practice, policy, and future research.	Sections 4.4 and 4.6 discuss cautious practice implications, the meaning of geographic practice variation, and the future-research agenda (Manuscript pp14-15).
**OTHER INFORMATION**			
Registration and protocol	24a	Provide registration information for the review, including register name and registration number, or state that the review was not registered.	Section 2.1 reports PROSPERO CRD420261451421 and the retrospective registration chronology (Manuscript p3).
	24b	Indicate where the review protocol can be accessed, or state that a protocol was not prepared.	The PROSPERO record is the accessible protocol record; Section 2 describes the implemented methods (Manuscript p3).
	24c	Describe and explain any amendments to information provided at registration or in the protocol.	Section 2.1 and 2.9 disclose the timing and the amendment from a three-study to ten-study threshold for funnel/Egger analyses; eligibility, outcomes, and primary random-effects approach were unchanged after registration (Manuscript pp3 and 6).
Support	25	Describe sources of financial or non-financial support for the review, and the role of the funders or sponsors in the review.	Funding statement (Manuscript p15).
Competing interests	26	Declare any competing interests of review authors.	Conflicts of Interest statement (Manuscript p16).
Availability of data, code and other materials	27	Report which of the following are publicly available and where they can be found: template data collection forms; data extracted from included studies; data used for all analyses; analytic code; any other materials used in the review.	Supplementary S2 provides study characteristics, and Supplementary S6 provides

PRISMA

PRISMA 2020 Checklist

Section and Topic	Item #	Checklist item	Location where item is reported
			study-level numerical inputs for all seven pooled outcomes. The Data Availability Statement and the S2/S6 ReadMe sheets identify the remaining unavailability of complete analytical code and ordinal-conversion flags (Manuscript p. 16; Supplementary S2; Supplementary S6).

From: Page MJ, McKenzie JE, Bossuyt PM, Boutron I, Hoffmann TC, Mulrow CD, et al. The PRISMA 2020 statement: an updated guideline for reporting systematic reviews. BMJ 2021;372:n71. doi: 10.1136/bmj.n71. This work is licensed under CC BY 4.0. To view a copy of this license, visit https://creativecommons.org/licenses/by/4.0/
